# Supplementary material for: Intraspecific variation in immune gene expression and heritable symbiont density
Source: PLoS Pathog. 2021 Apr 26;17(4):e1009552. doi: 10.1371/journal.ppat.1009552 (PMC8102006; doi:10.1371/journal.ppat.1009552)
Supplement: S1 Table — Numbers show log2 fold changes of expression in aphids with vs. without Regiella. Host genotype and symbiont strain are indicated in the header. (DOCX) [file ppat.1009552.s001.docx]

**S1 Table:** Significantly differentially expressed genes from the RNAseq experiments. Numbers show log_2_ fold changes of expression in aphids with vs. without *Regiella.* Host genotype and symbiont strain are indicated in the header.

| **Gene ID** | **Annotation** | **LSR**  **+ .LSR** | **LSR**  **+ .313** | ***Lotus***  **+ .313** | ***Ononis***  **+ .313** | ***Trifolium***  **+ .313** |
| --- | --- | --- | --- | --- | --- | --- |
| **ACYPI061678** | plexin A | 0.03 | -1.79 * | -1.19 * | -0.86 | -0.41 |
| **ACYPI44738** | plexin A1-like † | -0.19 | -3.09 * | -2.13 * | -2.48 | -0.46 |
| **ACYPI003478** | Hemocytin | -0.37 | -2.24 * | -1.94 * | -2.75 | -0.78 |
| **ACYPI004484** | Phenoloxidase 1 (subunit A3) | -1.40 | -3.25 * | -1.68 * | -2.06 | -0.14 |
| **ACYPI009767** | uncharacterized protein | -- | -- | 0.60 * | 0.16 | -- |
| **ACYPI008883** | probable vesicular glutamate transporter eat-4 † | -0.13 | -1.89 * | -1.78 * | -1.71 | -0.05 |
| **ACYPI53900** | Uncharacterized protein | -0.34 | 0.09 | -2.04 * | -1.67 | -0.45 |
| **ACYPI008487** | Apolipoprotein D | 0.03 | -2.08 * | -0.83 | -1.04 | -0.50 |
| **ACYPI006183** | Uncharacterized protein | 0.17 | -1.92 * | -0.88 | -0.69 | -0.22 |
| **ACYPI072244** | Phenoloxidase 2 (subunit 2) | -1.38 | -3.87 * | -1.56 | -2.08 | -0.20 |
| **ACYPI001483** | Echinoderm microtubule-associated protein-like 1 | -0.76 | -1.25 * | -0.49 | -0.45 | -0.18 |
| **ACYPI001736** | Uncharacterized protein | 0.09 | -1.09 * | -0.33 | -0.64 | -0.15 |
| **ACYPI50923** | Fibulin-1 † | -0.22 | -1.52 * | -0.62 | -1.12 | -0.37 |
| **ACYPI009930** | Uncharacterized protein | -0.77 | -2.13 * | 0.09 | -- | -0.12 |
| **ACYPI007618** | Neprilysin-11-like † | 0.37 | -1.08 * | -0.64 | -0.88 | -0.21 |
| **ACYPI007421** | Uncharacterized protein | 0.31 | -1.44 * | -0.57 | -0.88 | -0.36 |
| **ACYPI001380** | Cys-loop ligand-gated ion channel subunit-like / Neuronal acetylcholine receptor subunit | 0.06 | -1.28 * | -0.60 | -0.79 | -0.71 |
| **ACYPI061541** | Uncharacterized protein | 0.74 | 2.71 * | -1.63 | -2.10 | -0.51 |
| **ACYPI001359** | SPARC (Secreted protein acidic and rich in cysteine) | -0.42 | -0.71 * | -0.69 | -1.05 | -0.54 |
| **ACYPI24889** | Toll-like receptor 7 † | 0.01 | -1.35 * | -1.08 | -1.26 | -0.20 |
| **ACYPI000953** | Hydroxysteroid dehydrogenase-like protein 2 | -0.69 | -0.76 * | -2.63 | -0.76 | -0.13 |
| **ACYPI47960** | MD-2 related lipid-recognition protein-like † | -0.76 | -1.18 * | -0.39 | -0.52 | -0.21 |

* FDR < 0.05

† Uncharacterized in aphid genome v.2; annotation based on blast results.
