# Supplementary material for: Intraspecific variation in immune gene expression and heritable symbiont density
Source: PLoS Pathog. 2021 Apr 26;17(4):e1009552. doi: 10.1371/journal.ppat.1009552 (PMC8102006; doi:10.1371/journal.ppat.1009552)
Supplement: S2 Table — The top section labeled “Regiella” shows the results of statistical analyses of lines harboring Regiella. The bottom section labeled “Other Symbiont Species” shows the results of statistical analyses of lines harboring one of the Hamiltonella strains, Spiroplasma, or Serratia. Statistical significance at p < 0.05, p < 0.01, and p < 0.001 is indicated with a *, **, or *** respectively. (DOCX) [file ppat.1009552.s002.docx]

**S2 Table**: Results of post-hoc tests (Tukey’s HSD) analyzing gene expression of PO1 and Hemocytin (corresponding with Figures 1D and 1E). The top section labeled “*Regiella*” shows the results of statistical analyses of lines harboring *Regiella*. The bottom section labeled “Other Symbiont Species” shows the results of statistical analyses of lines harboring one of the *Hamiltonella* strains, *Spiroplasma*, or *Serratia*. Statistical significance at p < 0.05, p < 0.01, and p < 0.001 is indicated with a *, **, or *** respectively.

*Regiella*:

| ***PO1*** | **difference** | **Lower bound** | **Upper bound** | **Adjusted p-value** |
| --- | --- | --- | --- | --- |
| .313 vs No Symb | 2.98 | 1.44 | 4.53 | 0.0011 ** |
| .LSR vs No Symb | 1.53 | -0.01 | 3.08 | 0.0513 |
| .LSR vs .313 | -1.45 | -2.99 | 0.09 | 0.0652 |

| **Hemocytin** | **difference** | **Lower bound** | **Upper bound** | **Adjusted p-value** |
| --- | --- | --- | --- | --- |
| .313 vs No Symb | 2.54 | 1.61 | 3.47 | < 0.001 *** |
| .LSR vs No Symb | 1.03 | 0.10 | 1.96 | 0.031 * |
| .LSR vs .313 | -1.51 | -2.44 | -0.57 | 0.004 ** |

Other Symbiont Species:

| ***PO1*** | **difference** | **Lower bound** | **Upper bound** | **Adjusted p-value** |
| --- | --- | --- | --- | --- |
| *Hamiltonella* .179 vs *Spiroplasma* | 3.36 | 2.57 | 4.15 | < 0.001 *** |
| *Hamiltonella* .445 vs *Spiroplasma* | 3.47 | 2.68 | 4.26 | < 0.001 *** |
| *Serratia* vs *Spiroplasma* | 0.37 | -0.34 | 1.07 | 0.54 |
| No Symb. vs *Spiroplasma* | -0.18 | -0.97 | 0.61 | 0.96 |
| *Hamiltonella* .445 vs *Ham.* 179 | 0.10 | -0.76 | 0.97 | 0.99 |
| *Serratia* vs.  *Hamiltonella* .179 | -3.00 | -3.79 | -2.21 | < 0.001 *** |
| No Symb. vs  *Hamiltonella* .179 | -3.54 | -4.41 | -2.68 | < 0.001 *** |
| *Serratia* vs  *Hamiltonella* .445 | -3.10 | -3.89 | -2.31 | < 0.001 *** |
| No Symb. vs  *Hamiltonella* .445 | -3.65 | -4.51 | -2.78 | < 0.001 *** |
| No Symb. vs *Serratia* | -0.54 | -1.34 | 0.24 | 0.27 |

| **Hemocytin** | **difference** | **Lower bound** | **Upper bound** | **Adjusted p-value** |
| --- | --- | --- | --- | --- |
| *Hamiltonella* .179 vs *Spiroplasma* | -0.29 | -0.99 | 0.41 | 0.73 |
| *Hamiltonella* .445 vs *Spiroplasma* | -0.79 | -1.50 | -0.09 | 0.02 * |
| *Serratia* vs *Spiroplasma* | 0.03 | -0.59 | 0.66 | 0.99 |
| No Symb. vs *Spiroplasma* | -0.29 | -0.99 | 0.42 | 0.74 |
| *Hamiltonella* .445 vs *Ham.* 179 | -0.50 | -1.27 | 0.27 | 0.33 |
| *Serratia* vs.  *Hamiltonella* .179 | 0.33 | -0.38 | 1.03 | 0.64 |
| No Symb. vs  *Hamiltonella* .179 | 0.00 | -0.77 | 0.78 | 1.00 |
| *Serratia* vs  *Hamiltonella* .445 | 0.83 | 0.12 | 1.53 | 0.02 * |
| No Symb. vs  *Hamiltonella* .445 | 0.50 | -0.27 | 1.28 | 0.32 |
| No Symb. vs *Serratia* | -0.32 | -1.02 | 0.39 | 0.65 |
