# Supplementary material for: Intraspecific variation in immune gene expression and heritable symbiont density
Source: PLoS Pathog. 2021 Apr 26;17(4):e1009552. doi: 10.1371/journal.ppat.1009552 (PMC8102006; doi:10.1371/journal.ppat.1009552)
Supplement: S3 Table — Statistical significance at p < 0.05, p < 0.01, and p < 0.001 is indicated with a *, **, or *** respectively. (DOCX) [file ppat.1009552.s003.docx]

**S3 Table:** Results of post-hoc tests (Tukey’s HSD) analyzing *Regiella* densities in F1 lines. Statistical significance at p < 0.05, p < 0.01, and p < 0.001 is indicated with a *, **, or *** respectively.

| **Comparison** | **difference** | **Lower bound** | **Upper bound** | **Adjusted p-value** |
| --- | --- | --- | --- | --- |
| 663 vs (663x317.A) | 0.91 | 0.02 | 1.84 | 0.056 |
| 663 vs (663x317.B) | 1.22 | 0.07 | 2.38 | 0.033 * |
| 663 vs (663x317.D) | 1.30 | 0.38 | 2.23 | 0.003 ** |
| 663 vs (317x663.A) | 1.45 | 0.52 | 2.37 | < 0.001 *** |
| 663 vs (317x663.B) | 1.19 | 0.38 | 2.00 | 0.002 ** |
| 663 vs 317 | 2.38 | 1.37 | 3.38 | < 0.001 *** |
| (663x317.A) vs (663x317.B) | 0.31 | -0.89 | 1.51 | 0.98 |
| (663x317.A) vs (663x317.D) | 0.39 | -0.58 | 1.37 | 0.84 |
| (663x317.A) vs (317x663.A) | 0.54 | -0.44 | 1.51 | 0.58 |
| (663x317.A) vs (317x663.B) | 0.28 | -0.58 | 1.15 | 0.94 |
| (663x317.A) vs 317 | 1.46 | 0.41 | 2.52 | 0.003 ** |
| (663x317.B) vs (663x317.D) | 0.08 | -1.11 | 1.28 | > 0.99 |
| (663x317.B) vs (317x663.A) | 0.23 | -0.97 | 1.42 | > 0.99 |
| (663x317.B) vs (317x663.B) | -0.03 | -1.14 | 1.08 | > 0.99 |
| (663x317.B) vs 317 | 1.15 | -0.11 | 2.41 | 0.088 |
| (663x317.D) vs (317x663.A) | 0.14 | -0.83 | 1.12 | > 0.99 |
| (663x317.D) vs (317x663.B) | -0.11 | -0.98 | 0.76 | > 0.99 |
| (663x317.D) vs 317 | 1.07 | 0.02 | 2.13 | 0.045 * |
| (317x663.A) vs (317x663.B) | -0.25 | -1.11 | 0.61 | > 0.99 |
| (317x663.A) vs 317 | 0.93 | -0.13 | 1.98 | 0.11 |
| (317x663.B) vs 317 | 1.18 | 0.23 | 2.14 | 0.009 ** |
