# Supplementary material for: Intraspecific variation in immune gene expression and heritable symbiont density
Source: PLoS Pathog. 2021 Apr 26;17(4):e1009552. doi: 10.1371/journal.ppat.1009552 (PMC8102006; doi:10.1371/journal.ppat.1009552)
Supplement: S4 Table — Significance at p < 0.05, p < 0.01, and p < 0.001 is indicated with a *, **, or *** respectively. (DOCX) [file ppat.1009552.s004.docx]

**S4 Table**: Results of post-hoc tests (Tukey’s HSD) analyzing gene expression in F1 lines. Significance at p < 0.05, p < 0.01, and p < 0.001 is indicated with a *, **, or *** respectively.

| **Gene: *PO1*** | **Estimate** | **Std. Error** | **t value** | **Adjusted p-value** |
| --- | --- | --- | --- | --- |
| **Genotype-specific effect of *Regiella*:** | | | | |
| 663 | -2.49 | 0.59 | -8.64 | < 0.001 *** |
| 317 | 0.09 | 0.30 | 0.29 | 0.99 |
| (663 x 317) | -1.51 | 0.28 | -5.39 | < 0.001 *** |
| (317 x 663) | -0.68 | 0.28 | -2.42 | 0.13 |
| **Comparisons among interaction terms:** | | | | |
| 663 vs 317 | 2.58 | 0.42 | 6.12 | < 0.001 *** |
| 663 vs (663x317) | 0.99 | 0.40 | 2.45 | 0.095 |
| 663 vs (317x663) | 1.82 | 0.40 | 4.52 | < 0.001 *** |
| (663x317) vs 317 | 1.60 | 0.41 | 3.88 | 0.0039 ** |
| (317x663) vs 317 | 0.77 | 0.41 | 1.86 | 0.26 |
| (663x317) vs (317x663) | -0.83 | 0.40 | -2.10 | 0.18 |

| **Gene: *PO2*** | **Estimate** | **Std. Error** | **t value** | **Adjusted p-value** |
| --- | --- | --- | --- | --- |
| **Genotype-specific effect of *Regiella*:** | | | | |
| 663 | -2.72 | 0.28 | -9.61 | < 0.001 *** |
| 317 | 0.00 | 0.30 | <0.01 | >0.99 |
| (663 x 317) | -1.34 | 0.27 | -4.89 | < 0.001 *** |
| (317 x 663) | -0.73 | 0.27 | -2.65 | 0.09 |
| **Comparisons among interaction terms:** | | | | |
| 663 vs 317 | 2.72 | 0.41 | 6.64 | < 0.001 *** |
| 663 vs (663x317) | 1.38 | 0.39 | 3.50 | 0.014 * |
| 663 vs (317x663) | 2.00 | 0.39 | 5.06 | < 0.001 *** |
| (663x317) vs 317 | 1.34 | 0.40 | -3.32 | 0.02 * |
| (317x663) vs 317 | 0.72 | 0.40 | -1.80 | 0.37 |
| (663x317) vs (317x663) | 0.61 | 0.39 | 1.59 | 0.49 |

| **Gene: *Hemocytin*** | **Estimate** | **Std. Error** | **t value** | **Adjusted p-value** |
| --- | --- | --- | --- | --- |
| **Genotype-specific effect of *Regiella*:** | | | | |
| 663 | -2.08 | 0.41 | -5.10 | < 0.001 *** |
| 317 | -0.71 | 0.43 | -1.65 | 0.46 |
| (663 x 317) | -0.72 | 0.40 | -1.81 | 0.37 |
| (317 x 663) | -0.26 | 0.43 | -0.61 | 0.97 |
| **Comparisons among interaction terms:** | | | | |
| 663 vs 317 | 1.38 | 0.59 | 2.33 | 0.16 |
| 663 vs (663x317) | 1.37 | 0.57 | 2.41 | 0.14 |
| 663 vs (317x663) | 1.82 | 0.59 | 3.09 | 0.035 * |
| (663x317) vs 317 | 0.01 | 0.58 | 0.02 | > 0.99 |
| (317x663) vs 317 | -0.45 | 0.60 | -0.74 | 0.94 |
| (663x317) vs (317x663) | 0.46 | 0.58 | 0.78 | 0.92 |

| **Gene: *NOS*** | **Estimate** | **Std. Error** | **t value** | **Adjusted p-value** |
| --- | --- | --- | --- | --- |
| **Genotype-specific effect of *Regiella*:** | | | | |
| 663 | -0.53 | 0.53 | -1.00 | 0.84 |
| 317 | 1.24 | 0.55 | 2.25 | 0.18 |
| (663 x 317) | 0.29 | 0.51 | 0.56 | 0.98 |
| (317 x 663) | 0.82 | 0.51 | 1.60 | 0.48 |
| **Comparisons among interaction terms:** | | | | |
| 663 vs 317 | 1.77 | 0.77 | 2.31 | 0.16 |
| 663 vs (663x317) | 0.81 | 0.74 | 1.11 | 0.78 |
| 663 vs (317x663) | 1.35 | 0.74 | 1.83 | 0.36 |
| (663x317) vs 317 | 0.96 | 0.75 | 1.27 | 0.69 |
| (317x663) vs 317 | 0.42 | 0.75 | 0.56 | 0.98 |
| (663x317) vs (317x663) | 0.53 | 0.72 | 0.74 | 0.94 |
