# Supplementary material for: Intraspecific variation in immune gene expression and heritable symbiont density
Source: PLoS Pathog. 2021 Apr 26;17(4):e1009552. doi: 10.1371/journal.ppat.1009552 (PMC8102006; doi:10.1371/journal.ppat.1009552)
Supplement: S5 Table — Reg refers to Regiella insecticola; Ham refers to Hamiltonella defensa. (DOCX) [file ppat.1009552.s005.docx]

**S5 Table**: Collection information for the aphid genotypes used in this study. *Reg* refers to *Regiella insecticola*; *Ham* refers to *Hamiltonella defensa.*

| **Host Genotype**  **(lab code)** | **Location Collected** | **Year Collected** | **Original Symbionts** | **Biotype** |
| --- | --- | --- | --- | --- |
| LSR | Ithaca, NY, USA | 1998 | *Reg* | *Medicago sativa* |
| 317 | Glouchestershire, UK | 2003 | *Reg* | *Trifolium pratense* |
| 133 | Berkshire, UK | 2003 | *Ham* | *Ononis spinosa* |
| 663 | Oxfordshire, UK | 2014 | *None* | *Lotus corniculatus* |
