# Supplementary material for: Intraspecific variation in immune gene expression and heritable symbiont density
Source: PLoS Pathog. 2021 Apr 26;17(4):e1009552. doi: 10.1371/journal.ppat.1009552 (PMC8102006; doi:10.1371/journal.ppat.1009552)
Supplement: S6 Table — (DOCX) [file ppat.1009552.s006.docx]

**S6 Table**: Collection information for the symbiont strains used in this study.

| **Symbiont Genotype**  **(lab code)** | **Species** | **Location Collected** | **Year Collected** |
| --- | --- | --- | --- |
| .LSR | *Regiella insecticola* (clade 1) | Ithaca, NY, USA | 1998 |
| .313 | *Regiella insecticola* (clade 2) | Gloucestershire, UK | 2007 |
| .161 | *Spiroplasma* sp. | Oxfordshire, UK | 2006 |
| .179 | *Hamiltonella defensa* | Ithaca, NY, USA | 2015 |
| .445 | *Hamiltonella defensa* | Ithaca, NY, USA | 2015 |
| .509 | *Serratia symbiotica* | Knoxville, TN, USA | 2019 |
