# Supplementary material for: Intraspecific variation in immune gene expression and heritable symbiont density
Source: PLoS Pathog. 2021 Apr 26;17(4):e1009552. doi: 10.1371/journal.ppat.1009552 (PMC8102006; doi:10.1371/journal.ppat.1009552)
Supplement: S7 Table — (DOCX) [file ppat.1009552.s007.docx]

**S7 Table**: Primer Sequences

| Primer Target | F sequence (5’ to 3’) | | | R sequence (5’ to 3’) | | Citation | | |  |  |  |  |
| --- | --- | --- | --- | --- | --- | --- | --- | --- | --- | --- | --- | --- |
| **Screening for secondary symbiont infections:** | | | | | | | | | | | |  |
| *Regiella* | | AGTTTGATCATGGCTCAGATTG | | | GGTAACGTCAATCGATAAGCA | | | (1) | | |  |  |
| *Serratia* | | AGAGTTTGATCMTGGCTCAG | | | TTTGAGTTCCCGACTTTATCG | | | (1) | | |  |  |
| *Spiroplasma* | | ATTCTTCAGTAAAAATGCTTGGA | | | ACACATTTACTTCATGCTATTGA | | | (2) | | |  |  |
| *Hamiltonella* | | AGTTTGATCATGGCTCAGATTG | | | AAATGGTATTSGCATTTATCG | | | (1) | | |  |  |
| ***Regiella* MLST sequencing:** | | | | | | | | | | | | |
| accD | | CAYATGSGCATCTCTGCC | | | AATTCACTACTTTGAAAACCCGG | | | (1) | | |  |  |
| hrpA | | AAAACATTGTCTTCCGGG | | | TTTTCAAARTTNAGCAARTCMGG | | | (1) | | |  |  |
| ***Regiella* density via qPCR** | | | | | | | | | | | | |
| *Regiella* hrpA | | CGCATTGGGAGAAAAGCCAAG | | | CCTTCCACCAAGCCATGACG | | | This study | | |  |  |
| **Gene knockdown via RNAi** | | | | | | | | | | | | |
| lacZ | | **TAATACGACTCACTATAGGG**  AGACCACACCATGATTACGCCAAGCTC | | | **TAATACGACTCACTATAGGG**  AGACCACCATATCGGTGGTCATCATGC | | | (3) | |  |  |  |
| PO1  (ACYPI004484) | | **TAATACGACTCACTATAGGG**  CGAGCTACTGCGGTATCCTT | | | **TAATACGACTCACTATAGGG**  ACATTATTGGTGTTTGCGAATG | | | This study | |  |  |  |
| Hemocytin (ACYPI003478) | | **TAATACGACTCACTATAGGG**  TCGATCTTCGTCAACAATCA | | | **TAATACGACTCACTATAGGG**  AGGCCAACCTTGTTCTACTCC | | | This study | |  |  |  |
|  | | | ***T7 promoter sequence shown in bold** | | | | | | | | | |
| **Immune gene expression using qPCR:** | | | | | | | | | | | | |
| G3PDH  (ACYPI009769) | | CGGGAATTTCATTGAACGAC | | | TCCACAACACGGTTGGAGTA | | (4) | | | |  |  |
| NADH  (ACYPI009382) | | CGAGGAGAACATGCTCTTAGAC | | | GATAGCTTGGGCTGGACATATAG | | (4) | | | |  |  |
| β-tubulin  (ACYPI001007) | | GGCCAAGGGTCATTACACTGA | | | TGCGAACCACGTCCAACA | | (5) | | | |  |  |
| Rpl32  (ACYPI000074) | | CAAAGTGATCGTTATGACAAACTCAA | | | CGTCTTCGGACTCTGTTGTCAA | | (5) | | | |  |  |
| PO1  (ACYPI004484) | | CACTGTCCGTAGCATTGAT | | | GGCAGAATAATCGTGAGGTA | | (6) | | | |  |  |
| PO2  (ACYPI072244) | | ACGTGCGTATACGTTTCTCGAA | | | TGGCTTCCTATTCTGTTTTGCA | | This study | | | |  |  |
| Hemocytin (ACYPI003478) | | ACAATTCGGCGTAAAGGAGGT | | | TGGCATGTAATCGACGGTGT | | This study | | | |  |  |
| NOS  (ACYPI001689) | | TAGTGCTATCGGCAAACGGT | | | CGGATACTGCGGGAAGACAG | | This study | | | |  |  |

1. L. M. Henry *et al.*, Horizontally transmitted symbionts and host colonization of ecological niches. *Curr Biol* **23**, 1713-1717 (2013).

2. A. H. C. McLean *et al.*, Multiple phenotypes conferred by a single insect symbiont are independent. *Proc Biol Sci* **287**, 20200562 (2020).

3. B. J. Parker, J. A. Brisson, A Laterally Transferred Viral Gene Modifies Aphid Wing Plasticity. *Curr Biol* **29**, 2098-2103 e2095 (2019).

4. B. Li *et al.*, A large genomic insertion containing a duplicated follistatin gene is linked to the pea aphid male wing dimorphism. *eLife* **9** (2020).

5. S. H. Chung, B. J. Parker, F. Blow, J. A. Brisson, A. E. Douglas, Host and symbiont genetic determinants of nutritional phenotype in a natural population of the pea aphid. *Mol Ecol* 10.1111/mec.15355 (2020).

6. L. Xu, L. Ma, W. Wang, L. Li, Z. Lu, Phenoloxidases are required for the pea aphid's defence against bacterial and fungal infection. *Insect Mol Biol* **28**, 176-186 (2019).
