# Supplementary material for: Intraspecific variation in immune gene expression and heritable symbiont density
Source: PLoS Pathog. 2021 Apr 26;17(4):e1009552. doi: 10.1371/journal.ppat.1009552 (PMC8102006; doi:10.1371/journal.ppat.1009552)
Supplement: S8 Table — (DOCX) [file ppat.1009552.s008.docx]

**S8 Table**: Sequencing and alignment results

| **Host Genotype** | **Treatment** | **Read Pairs (after QC)** | **Map Rate** | **Read pairs mapped to an exon** |
| --- | --- | --- | --- | --- |
| LSR1 | Control A | 26,834,362 | L: 90.3%  R: 89.7% | 22,247,678 |
| LSR1 | Control B | 23,898,256 | L: 89.0%  R: 88.4% | 20,040,867 |
| LSR1 | Control C | 20,567,298 | L: 90.1%  R:89.5% | 17,513,945 |
| LSR1 | Control D | 30,839,554 | L: 90.5%  R: 89.4% | 26,232,845 |
| LSR1 | + Clade 1 (.LSR) A | 25,834,362 | L: 90.3%  R: 89.7% | 22,247,678 |
| LSR1 | + Clade 1 (.LSR) B | 22,145,457 | L: 89.1%  R: 88.1% | 18,466,505 |
| LSR1 | + Clade 1 (.LSR) C | 27,986,917 | L: 89.4%  R: 89.1% | 23,737,329 |
| LSR1 | + Clade 1 (.LSR) D | 21,490,058 | L: 89.8%  R: 89.3% | 18,248,263 |
| LSR1 | + Clade 2 (.313) A | 20,099,181 | L: 89.2%  R:88.5% | 16,863,392 |
| LSR1 | + Clade 2 (.313) B | 19,020,667 | L: 88.9%  R: 88.5% | 15,942,798 |
| LSR1 | + Clade 2 (.313) C | 21,612,263 | L: 89.7%  R: 89.0% | 18,295,757 |
| LSR1 | + Clade 2 (.313) D | 23,324,084 | L: 89.6%  R: 88.6% | 19,704,560 |
| *Lotus corniculatus* | Control A | 28,664,619 | L: 86.7%  R: 86.1% | 23,297,559 |
| *Lotus corniculatus* | Control B | 32,207,455 | L: 87.5%  R: 86.8% | 25,222,986 |
| *Lotus corniculatus* | Control C | 25,987,704 | L: 86.7%  R: 86.3% | 21,067,089 |
| *Lotus corniculatus* | *Regiella* A | 24,420,268 | L: 87.1%  R: 86.7% | 19,780,712 |
| *Lotus corniculatus* | *Regiella* B | 22,622,062 | L: 87.7%  R: 87.2% | 18,485,841 |
| *Lotus corniculatus* | *Regiella* C | 30,883,446 | L: 87.3%  R: 86.8% | 25,106,295 |
| *Ononis spinosa* | Control A | 25,428,345 | L: 87.6%  R: 86.9% | 20,652,853 |
| *Ononis spinosa* | Control B | 24,322,991 | L: 87.8%  R: 87.2% | 19,823,044 |
| *Ononis spinosa* | Control C | 30,277,729 | L: 87.7%  R: 87.2% | 24,579,747 |
| *Ononis spinosa* | *Regiella* A | 26,677,905 | L: 87.9%  R: 87.3% | 21,741,656 |
| *Ononis spinosa* | *Regiella* B | 29,108,115 | L: 87.8%  R: 87.3% | 23,498,863 |
| *Ononis spinosa* | *Regiella* C | 25,669,486 | L: 87.8%  R: 86.4% | 20,955,594 |
| *Trifolium pratense* | Control A | 22,440,188 | L:86.7%  R:85.9% | 17,919,579 |
| *Trifolium pretense* | Control B | 20,932,628 | L: 86.8%  R: 85.9% | 16,741,330 |
| *Trifolium pretense* | Control C | 22,905,550 | L: 86.6%  R: 85.6% | 18,228,176 |
| *Trifolium pretense* | *Regiella* A | 27,502,459 | L: 87.8%  R: 87.3% | 22,631,920 |
| *Trifolium pretense* | *Regiella* B | 20,253,813 | L: 87.0%  R: 86.7% | 16,395,529 |
| *Trifolium pratense* | *Regiella* C | 23,107,988 | L: 86.3%  R: 85.2% | 18,251,833 |
