# Supplementary material for: Intraspecific variation in immune gene expression and heritable symbiont density
Source: PLoS Pathog. 2021 Apr 26;17(4):e1009552. doi: 10.1371/journal.ppat.1009552 (PMC8102006; doi:10.1371/journal.ppat.1009552)
Supplement: Attachment — Submitted filename: Response_letter.pdf [file ppat.1009552.s010.pdf]

Please note that the line numbers below refer to the 'clean' version without tracked changes.

Reviewer #1: This manuscript focuses upon beneficial bacterial symbionts and their relationships with their aphid hosts, particularly from an immune modulation perspective. The bacterial symbionts provide benefits to the aphid hosts, but there may be competing interests on the part of the symbionts (for increased density and potential transmission) vs. the aphids (control of symbionts potentially harmful at high densities). These competing interests could be modulated by manipulation of the aphid immune system by either the aphids or the bacteria. This manuscript seeks to determine the genetic basis of any immune interactions by measuring gene expression differences in aphids with different strains of Regiella symbiont infections and also among different aphid genotypes. A small number of immune genes were found to change in expression, depending upon the symbiont type. The authors then knocked down expression of a few key immune genes to determine the impact upon symbiont density and found that one affects the other. Finally, the authors crossed some aphid genotypes together to determine how variation in genotype affected symbiont density and also immune gene expression. Their results showed that genotype influences both factors.

I found this manuscript very well put together. The feedback between beneficial symbionts and insect immune systems is a very interesting topic, and this work contributes new information on the impact of aphid immune gene expression upon symbiont density in hosts, showing that the aphid immune system is an important part of the relationship between insects and their symbionts.

- **Thanks to the reviewer for their valuable feedback on this manuscript.**

Insect phenoloxidase proteins are actually produced as pro-phenoloxidase enzymes that become activated to phenoloxidase by enzymatic cleavage. This means that their regulation primarily occurs at the post-transcriptional level by activation of a serine protease cascade induced by immune challenge. Therefore it is unclear what impact changes in the expression of the phenoloxidase genes will have upon aphid immunity. It would be very worthwhile to see how levels of phenoloxidase proteins change with different facultative symbiont infections and how much of the protein is in the active vs. inactive forms.

- **We agree it would be beneficial to have protein expression data for PO, and we've added in a caveat to the discussion acknowledging the reviewer's point about this limitation of our study, l. 300: "It is important to note that regulation of phenoloxidase also occurs at the post-translational level [60], and future work is needed to link changes in gene expression in the presence of symbionts to protein levels." We're not able to generate the mass spectrometry data needed to address this point for this revision.**

Was it possible to replicate experimental results? qPCR results are shown for a relatively small sample size, making it unclear if the same results would be obtained in a replicated experiment. It was not mentioned in the methods whether experiments were replicated and had qualitatively similar results, but it would be good to add this is possible.

- **We used qPCR in Figures 1D and 1E to demonstrate that Phenoloxidase and Hemocytin are downregulated in aphids harboring *Regiella* compared to symbiont-free individuals—a validation of our RNAseq data. Importantly, this same downregulation was replicated independently in a second experiment using additional aphid lines with both RNAseq and qPCR (Figures 3 & 4C). We therefore feel that this finding is well supported and replicated. Each data point in Figures 1D, 1E, and Figure 4 represents a separate aphid line in which we established an independent symbiont infection using injection (rather than establishing a symbiosis with a single injection and then splitting the line into biological replicates)—the difficulty of this procedure limits our sample size.**

I don't think there's enough evidence presented in this work to determine whether the differences in Regiella density observed between aphid lines is due to aphids manipulating their own immune responses or Regiella actively suppressing immune gene expression. As I was reading the results, I highlighted the word "suppressed" on line 285, and was surprised by the choice of word here rather than something more unbiased. When I reached the discussion I saw that the authors are making an argument that Regiella is responsible for manipulating host gene expression. Arguments related to high Regiella infection densities being maladaptive for aphid fitness (found in other studies) were used to support this conclusion. However, I wonder if the impact of Regiella upon fitness is also variable and correlated with frequencies of co-occurrence. How often does the LSR1 strain of aphids encounter Regiella from "clade 2"? Overall I found the arguments in the discussion somewhat confusing with respect to the data. The authors may be able to make their arguments clearer or perhaps treat both possibilities more equally.

- **We have revised the manuscript extensively to address this point. Specifically, we no longer refer to the decrease in immune gene expression we measure in this study as 'suppression,' except for in two instances at the end of the discussion after we discuss the evidence from other studies, and after the words "We propose that …" in the abstract.**

I find the indications of statistical significance in figures confusing, e.g. Figure 2A and B, Figure 4C. It is clear that there are drastic differences between .LSR and .313 strains for phenoloxidase expression 8 days after infection. Is the line and the asterisk at the top of the figure meant to convey this, or that there are differences between lacZ and PO1 treatments? I believe that all statistically significant differences in the data should be indicated, even if it isn't the main result that is being conveyed (within reason).

- **Sorry for the confusion. We've revised Figures 2A and 2B to show the differences between .LSR and .313 strains for PO expression at day 8 and differences in titer at both time points, as suggested. The new figure legend should now make clear that the indications of statistical significance at the top of the figures reflect differences among treatment groups, and those to the right of the figures reflect differences among *Regiella* strains.**
- **We've revised Figure 4C to include the within-genotype effects of *Regiella* on the expression of each gene; significance is shown with a vertical grey line and asterisks as appropriate.**

In figure 1D and E, I think the grey bars showing means are missing (they are in all other comparable figures)

- **Thanks, we've added these to the revised figure.**

References to figures are often incorrect in the text and should be corrected.

- **Thanks for catching this mistake; we have carefully checked all references to figures throughout the manuscript and believe these are correct in the revised version.**

Figure 1 and Figure 3 are confusing regarding statistical cut-offs used. It would be nice to see how the FDR cut-offs correspond to the pvalues shown on the y axes. It seems logically unideal to use a FDR <0.05 cut-off for all comparisons except those shown in Figure 3A.

- **In response to this point, we removed the elements of Figure 3 that referred to the higher FDR cutoff of < 0.1, and also removed any reference to this statistical cut-off from the text. (We did use the same cutoffs for both analyses in the original submission, i.e. we also considered genes with an FDR < 0.1 in the Figure 1 analysis but there are no genes that have an FDR value between 0.05 and 0.1 in this transcriptome set; using just the 0.05 cutoff for all 4 analyses is probably the clearest and best option). The colored circles in Figures 1 and 3 show the relationship between p-value and FDR (i.e. FDR < 0.05 for colored circles).**

How are the LSR1, Lotus, Ononsis, and Trifolium lines related (closely or distantly)?

- **Peccoud et al. (2009) PNAS performed a structure analysis of variation in microsatellite loci among biotypes, and based on these data they calculated pairwise genetic differentiation based on a standardized F_{SC}. There are some clear patterns of differentiation among biotypes; for example, aphids from *Lathyrus pratensis* (not included in our study) are highly divergent from other biotypes and may represent an incipient species. There is less genetic differentiation among the biotypes we focused on; *Trifolium* and *Medicago* are most closely related (33.1% differentiation based on standardized F_{SC}) and *Ononis* is the most distant from the other biotypes (69.1%, 66.2%, and 80.6% from *Trifolium*, *Lotus*, and *Medicago* respectively). We added a brief discussion of genetic distance to the revised results/discussion: "The genetic distance among these biotypes is variable, with *Trifolium* and *Medicago sativa* (LSR1) possibly the most closely related and *Ononis* the most distant; unlike some biotypes (e.g. *Lathyrus pratensis*), those included here are not thought to represent incipient species [29])." (l. 213)**

Reviewer #2: In many host-symbiont relationships, there is variation across a population or populations in the titer of symbionts. However, the mechanisms that regulate these titers are not fully understood, particularly considering beneficial vs harmful symbionts. Here, Nichols et al. demonstrate that a strain of Regiella symbionts in aphids can increase its density through suppression of host immunity. This trait varies across host genetic backgrounds, potentially indicating that there is an arms race of adaptation and counter-adaptation between aphid and symbiont across populations and strains. This is a novel study in that it is one of the few to identify a mechanism of heritable symbiont regulation, and the mechanism is itself novel. The experiments are well executed with a strong design, the figures are visually appealing, and it is clearly written.

- **We thank the reviewer for their helpful feedback on our manuscript.**

There are several minor questions and suggestions that would help enhance the paper if included in the text:

-Line 129: It would be beneficial to give a little more background information on the 313 strain here in the main text including host background, host plant, and collection location as an easy comparison to the lsr strain

- **This information has been added to the revised text.**

-Figure 1D: Both of the Hamitonella strains also reduce phenoloxidase 1 at levels comparable to 313; are they also known to have relatively high densities?

- **Unfortunately, we do not know anything about the density of these *Hamiltonella* strains, but exploring the effects of other species and strains of facultative symbionts on aphid immune gene expression is part of our plans for future work.**

-Line 165: It would help to briefly mention the rationale for using these two genes in particular as opposed to the other differentially expressed genes for rnai and qpcr experiments

- **We present the data in this manuscript in a slightly different order than we carried out the experiments. Out of the immune genes identified in our Figure 1 transcriptome, we chose PO1 and hemocytin for the qPCR and RNAi work in Figures 1 and 2 because we found that they were also significantly downregulated in the *Lotus* line in Figure 3, and thus appeared to be the best candidate genes for functional work. We explain this in the revised Methods, as suggested (l. 398).**

-Line 170: In this section, it may help to mention/compare the tissue localization of these microbes for naïve readers (all the same or are there differences?)

- **Thanks, we've added a few sentences about tissue localization of the different aphid facultative symbionts starting at line 162: "Aphid symbionts in the family Enterobacteriaceae (*Regiella, Hamiltonella,* and *Serratia*) live both in hemolymph and inside of the insect cells (reviewed in [52]). In contrast, *Spiroplasma* is mainly extracellular in most insects, and has not been identified inside of aphid cells [53]. Qualitatively, then, our results do not suggest that differences in immune gene expression among facultative symbiont species are due to localization in different tissues."**

-All figures: It would help to name the statistical tests and * values for all figures within the captions

- **Edited as suggested.**

-Figure 3: How closely related are the different aphid host backgrounds tested in this study- is there a pattern in relation to the Regiella densities? Which Regiella clades do these other host genotypes naturally harbor- are they all clade 2?

- **Re: relatedness among aphid biotypes. Please see our response to reviewer 1, and the associated changes to the manuscript.**
- **To address the question about biotype-*Regiella* clade patterns, we've added in more info about the tight association between *Trifolium* aphids and Clade 2 *Regiella* to the introduction (starting at l. 103), and we've highlighted the collection info and original symbiont compliment of each aphid line used in the experiments in the main text (e.g. l. 129 and l. 210-213).**

-Line 286: Italicize/capitalize Lotus

- **Fixed**

-Figure 4: What is meant by the letters at the bottom of the panels by the X axes/genotypes? I could not find the information in the caption. Why only two groups for trifolium x lotus in B vs three groups for the inverse?

- **These letters refer to the F1 line for that cross. The difference between two and three lines for each direction of the cross in Figure 4B is simply because that's how many eggs we were able to hatch. We have made it clear in the revised figure legends what these letters refer to (we've kept them in this paper because we are using these lines in current work and think it will be helpful to make this and our future papers comparable).**

-Figure 4B: Italicize Regiella on Y axis

- **Fixed**

-Figure 4C: Perhaps I missed this, but were statistics run to compare the expression differences within each genotype? Right now the stats are across genotypes, which is a helpful comparison, and the individuals with or without Regiella are differentiated visually. But, is there statistically a difference in gene expression within each genotype between Regiella vs no symbiont, and how do these differences compare across the lines? Visually, I can appreciate the trend, but the statistics would be helpful as well.

- **We had not reported the specific effects of *Regiella* within each genotype for the data in Figure 4 but can see the value of doing so; we have performed these analyses for the revised version and include the statistical results in Table S4. We also depict the results of these tests visually in figure 4C (see response to reviewer 1, above).**

-It is interesting that the .313 Regiella and Trifolium type host were collected in the same place and the Trifolium type had clade 2 symbionts already, and that the Lotus type was collected with no symbiont. I found this information in the supplement, but I think it would be beneficial to directly mention this correlation in the discussion to bolster the adaptation-counteradaptation argument.

- **Collection info has been added to the main text in the revised version, as suggested.**

Reviewer #3: Nichols et al., present we well written report on the interaction between secondary symbionts of pea aphids and the host immune system. Specifically, using a thorough set of experiments, the authors clearly demonstrate the effect of Regiella secondary symbiont on the expression and function of several specific aphid immune genes and resultant symbiont titer. The strength of this work derives from the set of experiments that address their basic question through the cross-infection of aphid lines with different strains of Regiella to identify host immune genes that underlie the control of symbiont titer, experimental infection of aphid biotypes that have different host genotypes with Regiella strains to show specific host-symbiont genotype interactions, and the generation of hybrid lines to demonstrate that the trends are genetically controlled and heritable. Moreover, the authors further use RNAseq, RNAi, and qPCR validation to show the specific actions of immune genes in modulating symbiont titer. The authors propose that some Regiella strains are able to antagonistically increase their titer in some host genotypes through the suppression of certain host immune response genes (Phenoloxidase and Hemocytin). The authors interpret this as antagonistic co-evolution between a “beneficial” symbiont and the host

Overall, I found the presented work to be well done, well written, and very compelling. The manuscript is easy to read and clearly outlines the purpose, results, and implications of the work done. The figures are clear and demonstrate the results very well. I have only a few relatively minor comments that may be considered (through discussion) prior to publication:

- **Thanks to the reviewer for their useful feedback**

1) Since the authors infer an antagonistic coevolution model, it might be worthwhile to add a bit more specific detail to the Introduction framing the antagonism between Regiella and aphid hosts. The fungal protection and general theory of host-symbiont antagonism is addressed here, but some introductory material setting the specific basis for the theory/hypothesis that drives this work would help the reader understand the complicated Regiella-aphid interaction better.

- **To address this point, we have added in more information about the aphid-*Regiella* system to the introduction as suggested, e.g. l. 103.**

2) The authors reasonably logic that certain Regiella strains are the causative agent of suppressed immune response in some aphids. But, is there any information that can be gleaned from strain specific genomes to indicate, or permit speculation of, the specific bacterial mechanisms?

Alternatively, the authors collected a significant amount of RNAseq data from these systems. Could that data be mined to identify highly expressed and/or differentially expressed bacterial genes across their infection studies to hypothesize what mechanism are at play.

- **We very much want to know more about the microbial mechanisms that alter immune gene expression (and protection against fungus), and we are working to generate genomic and functional genetic**

resources for studying this microbe. But as of now, we don't have much of a genome sequence for strain .313 and thus can't compare it to the .LSR strain. Further, we've tried in other studies to generate transcriptome data for *Regiella* out of aphid samples, and have found that *Regiella* transcripts are extremely rare, even when we try to deplete eukaryotic and prokaryotic rRNA or specifically select for symbiont transcripts. Based on these observations and that our methods included poly-A selection during library prep, we don't expect there to be many *Regiella* transcripts to analyze for differential expression across aphid genotypes.

3) In figure 1D, it looks as though both Regiella strains causes decline in PO1. The biological significance of this is not well explained, nor discussed. I see that the effect of .313 is apparently stronger, but the other strain also seems to have a strong effect here. Is the ability of Regiella .313 to establish higher titers due to the bulk suppression of both PO1 and Hemocytin? Perhaps some clarifying points in the discussion can help put this together more strongly.

- **We did not find a statistically significant effect of harboring .LSR on PO1 expression from qPCR or from RNAseq. We also find that downregulation of hemocytin is statistically significantly stronger in the presence of strain .313 than strain .LSR. One possibility, as the reviewer suggests, is that the stronger differential expression of immune genes by strain .313 vs .LSR explains its higher density; we mention this in the revised discussion (l. 261).**

It also appears that other secondary symbionts have the same effect in suppressing PO1, but not-necessarily Hemocytin. Does this provide any insight on the combinatorial effect of Regiella .313 on suppressing both genes? Is it also known whether Hamiltonella and Regiella increase their titer through modulation of PO1? Some discussion here could be brought into the general antagonistic co-evolution theory, as well.

- **This hasn't been investigated in *Hamiltonella*. Of the secondary symbionts we investigated for effects on PO1 and hemocytin expression, only *Hamiltonella* had an effect on PO1 expression—so with this one exception, the other secondary symbionts we investigated generally did not have the same effect as *Regiella* on immune gene expression. We think it is probably premature to speculate on whether these same effects on density would also occur in *Hamiltonella* – our goal with this part of the study was to determine if other symbionts that are protective against fungus (*Spiroplasma* but not *Hamiltonella* or *Serratia*) also downregulate immune gene expression.**

4) For the RNAi experiments illustrated Figs 2A-B, it seems that the lacZ control has a similar effect, or at least trend, as the PO1 dsRNA target and resultant Regiella titer… particularly over the long-term. This result is not really discussed. Does simply injecting the aphids, or the buffer used, cause a response in PO1 expression system with downstream effects on Regiella titer? Were any other controls used in this work, or in previous work, to verify the effect of RNAi delivery on the immune system response?

- **The objective of using lacZ is specifically to control for the effects of the injection, the buffer used to dissolve the dsRNA, and exposure to dsRNA and therefore overall to isolate the specific effects of PO1 knockdown. The reviewer is right that the stab could influence immune gene expression compared to unstabbed aphids, though this effect will be consistent across the lacZ and PO1 treatments. We've added in a caveat to this effect in the revised manuscript (l. 173).**

5) Finally, the antagonistic co-evolution hypothesis is not revisited in the discussion. It might be nice to summarize the results presented here in the broad theory as it pertains to this body of theory, insect immune system evolution, and host-symbiont interactions.

- **We expand on this idea in the revised discussion: "Our findings might more broadly suggest, then, that hosts and beneficial heritable microbes can evolve antagonistically, which has been suggested by other studies in aphids [9, 58] and other organisms [59]." (l. 292). The changes suggested above with respect to 'immune suppression' by *Regiella* vs. accommodation of *Regiella* by hosts, have made us hesitant to over-interpret our results in terms of antagonistic evolution.**
